# Combined Papillary Renal Cell Carcinoma with Neuroendocrine Differentiation and Mucinous Tubular and Spindle Cell Carcinoma

**DOI:** 10.1155/2018/8734823

**Published:** 2018-12-31

**Authors:** Gang Wang, Ren Yuan, Tracy Tucker, Allan B. Gates, Christopher D. Bellamy, Malcolm M. Hayes

**Affiliations:** ^1^Department of Pathology, BC Cancer Vancouver Centre, Vancouver, BC, Canada; ^2^Department of Radiology, BC Cancer Vancouver Centre, Vancouver, BC, Canada; ^3^Department of Pathology, Abbotsford Regional Hospital, Abbotsford, BC, Canada; ^4^North Island Pathology, Comox, BC, Canada

## Abstract

A unique case of combined papillary renal cell carcinoma (PRCC) and mucinous tubular and spindle cell carcinoma (MTSCC) presenting in a man aged 67 years is reported. The two separate components were distinct on morphological, immunohistochemical (IHC), and genetic grounds, while type 2 PRCC predominated. Three years after the initial diagnosis, the PRCC component metastasized to the lungs where it morphologically mimicked a pulmonary neuroendocrine tumor. Retrospectively focal neuroendocrine differentiation was demonstrated by IHC in the PRCC component of the primary neoplasm.

## 1. Introduction

PRCC and MTSCC constitute, respectively, 18% and <1% of all renal tumors [1]. They show some overlap in morphology and immunohistochemical profiles, but are genetically distinct. Renal cell carcinoma (RCC) with neuroendocrine differentiation has been rarely reported. Chromophobe renal cell carcinoma (ChRCC) with neuroendocrine differentiation and/or a neuroendocrine-like pattern has been described and characterized [2–5]. Rare cases of PRCC are reported showing positivity for neuroendocrine markers [6]. This report describes a PRCC with neuroendocrine differentiation combined with a component of MTSCC. The metastasis to lung mimicked a neuroendocrine tumor posing diagnostic confusion.

## 2. Case Presentation

**Figure 1:**
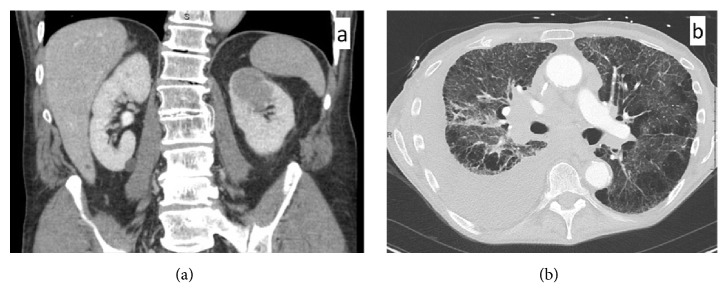
(a) Contrast enhanced CT shows a 4.5 cm left upper pole renal mass. (b) Chest CT shows an ill-defined obstructive right perihilar mass, which is inseparable from the extensive mediastinal and hilar lymphadenopathy. There is irregular and nodular inter-/intra-lobular septal thickening predominantly in the right middle and lower lobe with ipsilateral pleural effusion, suggestive of lymphangitic carcinomatosis.

A 67-year-old man with a significant smoking history presented with a 4.5 cm enhancing left upper pole renal mass detected on CT scan and treated by radical nephrectomy (Figure 1(a)). Three years later, he presented with a cough and shortness of breath. A chest CT showed an obstructive central mass associated with distal atelectasis/consolidation and moderate right pleural effusion. There was bilateral extensive mediastinal and hilar lymphadenopathy, and irregular inter-/intra-lobular septal thickening predominantly involving the right middle and lower lobe suggesting lymphangitic carcinomatosis (Figure 1(b)). CT of the upper abdomen at the same time showed no new mass at left renal bed or in the right kidney.

**Figure 2:**
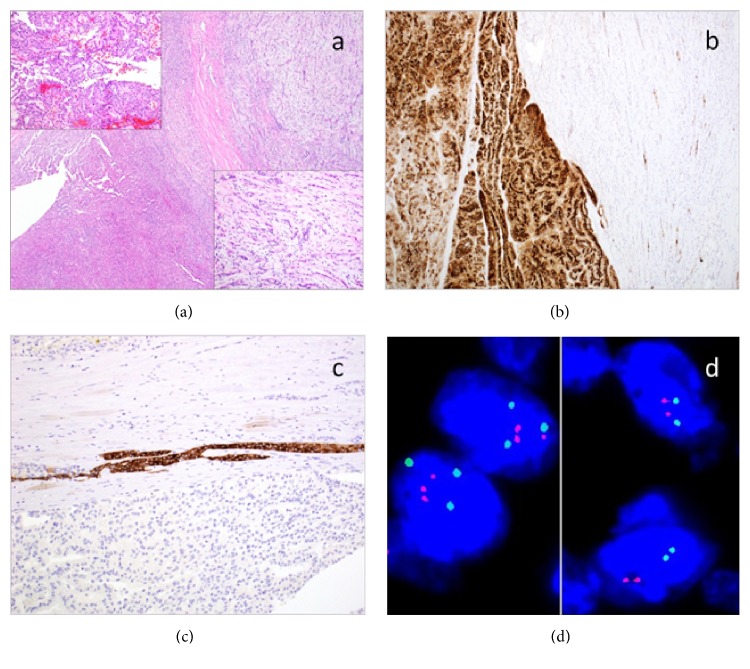
Photomicrographs of the primary kidney tumour. (a) There were two distinct morphologies. The main tumour showed a prominent papillary structure, eosinophilic cytoplasm, Fuhrman grade 3 nuclei (left). The smaller focus of tumour showed long tubular profiles or cord-like growth pattern of uniform, bland, low cuboidal cells with eosinophilic, focally vacuolated cytoplasm which transition to anastomosing spindle cells, with stroma showing myxoid and bubbly with abundant extracellular mucin (right). (b) The PRCC component showed positive staining for CD10 (left). The MTSCC component was negative for CD10 (right). (c) The PRCC component had small foci positive for synaptophysin. (d) Fluorescent in situ hybridization analysis showing three centromere 17 signals consistent with trisomy 17 in the PRCC component (left, green dots), while the MTSCC component was negative for trisomy 17 (right, green dots).

Gross examination revealed a gray-white, circumscribed, encapsulated, focally necrotic mass measuring 4.8 cm in largest dimension in the superior pole of the kidney. The tumor focally invaded perinephric tissues but was completely resected. Microscopically, the majority (95%) of the tumor showed the morphology of a Type 2 PRCC with a prominent papillary architecture. The cells were polygonal in shape and exhibited abundant eosinophilic granular cytoplasm and Fuhrman grade 3 nuclei (Figure 2(a), left). IHC showed positive staining for CK7, Racemase, and CD10 (Figure 2(b)). Additional IHC performed in retrospect, showing that a small focus of PRCC component was strongly positive for synaptophysin (Figure 2(c)) but negative for CD56 and chromogranin, indicating a neuroendocrine differentiation. A minor component (5%) of the tumor showed features of MTSCC (Figure 2(a), right). This component exhibited elongated tubules and cords of uniform, bland, low cuboidal cells with eosinophilic, focally vacuolated cytoplasm and transitions to anastomosing spindle cells. The stroma was myxoid with abundant extracellular mucin. IHC showed this component of the tumor was focally positive for CK7 and Racemase, but negative for CD10, synaptophysin, CD56, and chromogranin. Fluorescent in situ hybridization (FISH) analysis demonstrated no evidence of aneuploidy for chromosome 7 in either tumor area. The nuclei in the PRCC component had three centromere 17 signals consistent with trisomy 17, those in the MTSCC component were negative for trisomy 17 (Figure 2(d)).

**Figure 3:**
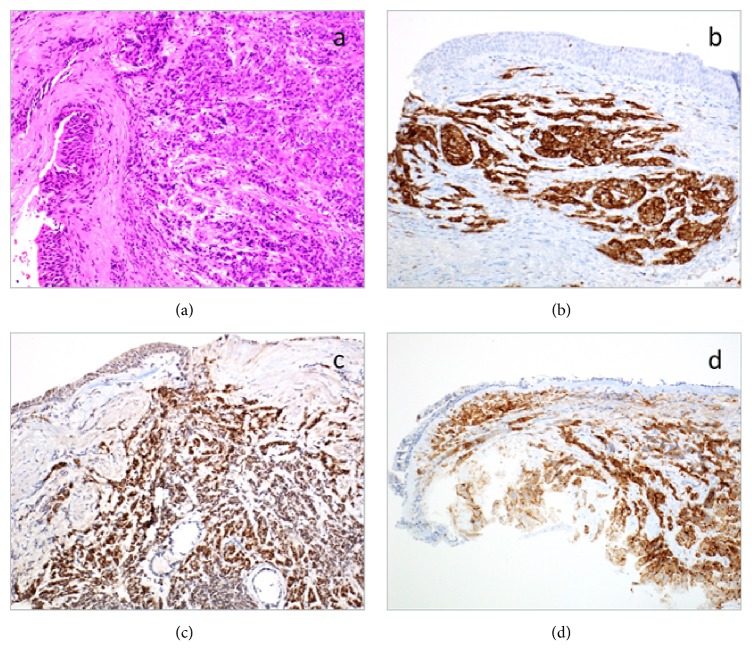
Photomicrographs of the lung metastasis. (a) A very infiltrative tumour in the submucosa of the bronchus with a nested and trabecular architecture and no definite papillary architecture. (b) The tumour was diffusely positive for synaptophysin. (c) The tumour was strongly positive for PAX8. (d) The tumour showed positive staining for CD10.

The recent transbronchial biopsy showed an infiltrative tumour with nested and trabecular architecture but no papillary component. Nests and cords of small polygonal cells were surrounded by delicate fibrovascular stroma. The cells had a moderate amount of vacuolated, granular eosinophilic cytoplasm (Figure 3(a)) suggestive of an endocrine neoplasm. Mitoses were quite numerous. The nuclei were medium-sized, many showing a central nucleolus, but lacked the “salt-and-pepper” chromatin pattern typically seen in neuroendocrine tumors. However, IHC showed an endocrine profile, diffusely positive for synaptophysin (Figure 3(b)) and focally for chromogranin and CD56. The tumor was also strongly positive for pan-cytokeratin, renal cell carcinoma antigen (RCC), PAX8 (Figure 3(c)), and CD10 (Figure 3(d)), but was negative for TTF1, which supported the diagnosis of a metastatic PRCC with neuroendocrine differentiation. The Ki-67 immunostain showed a proliferative index of 25%.

## 3. Discussion

PRCC is thought to be derived from the renal tubular epithelium [7]. It is categorized into two subtypes: papillary renal cell carcinoma type 1 (PRCC1) and type 2 (PRCC2) [1, 8]. PRCC1 usually presents as a well circumscribed and encapsulated mass composed of papillae lined by a single layer of cuboidal epithelial cells with Furhman Grade 1 or 2 nuclei and scanty pale cytoplasm. Aggregates of foamy macrophages in the background stroma of the papillae are a common finding. Necrosis is rare in PRCC1. PRCC2 demonstrates a varied cytomorphology, often having abundant eosinophilic cytoplasm, more pleomorphic nuclei, and prominent nucleoli. It often shows diffuse infiltration of the peri-tumoral tissues. Necrosis and nuclear pseudo-stratification are common in PRCC2. By IHC, PRCC often shows expression of EMA, Racemase, RCC, Vimentin, and CD10, which may help to distinguish it from other RCC subtypes [1]. Strong, homogeneous CK7 expression is more frequent in PRCC1 than in PRCC2 [1]. PRCC2 probably encompasses one or more subtypes of aggressive RCC with a poorer outcome than PRCC1 [9]. Genetic analysis of PRCC typically demonstrates chromosomal gain, most often of chromosomes 7 and 17, present in 68-75% and 67-80% of PRCCs, respectively. Trisomy 7 is also a common finding in several other human cancers including 18-30% of non-PRCCs, normal renal cells, and several benign conditions. Therefore, trisomy 7 is not specific for PRCC. In contrast, non-random gain of chromosome 17 is uncommon in other forms of RCC (present in 2.6% of CCRCCs) and other human cancers and is quite specific for PRCC [10].

MTSCC is an unusual renal carcinoma of probable distal nephron differentiation with prominent spindle cell change and a myxoid stroma. The histologic spectrum and IHC profile are variable. MTSCC is characterized by an elongated tubular and cord-like architecture, cuboidal to spindled cells with low nuclear grade, and a myxoid/mucinous stroma [1]. IHC shows uniform expression of CK7 and AMACR suggesting its proximal nephron origin and intimate relationship to PRCC [11]. Emerging evidence suggests that MTSRCC is a histologically heterogenous and shows morphologic and immunohistochemical features overlapping with papillary RCC, but it remains a genetically distinct entity. FISH analyses have shown consistently that MTSRCC lacks of the gains of chromosomes 7 and 17 and losses of chromosome Y that characterise PRCC [12]. Although some cases of MTSRCC and PRCC have overlapping morphology, typically, PRCC contains complex branching papillae, the reverse of MTSRCC. Although PRCC and MTSRCC share a CK7 and AMACR positive profile, MTSRCC is usually negative for CD10 while PRCC is positive [11]. In our case, the two components (PRCC and MTSRCC) were morphologically distinct, had different CD10 staining patterns and genetic features, and were present side by side within the same tumour mass. To the best of our knowledge, it is the first case report of a combined PRCC and MTSRCC.

The tumour that presented in the lung/mediastinum three years later posed a diagnostic challenge. While the cells lacked the “salt-and-pepper” chromatin pattern typically seen in neuroendocrine tumours, the architecture, vascular pattern, and eosinophilic granular cytoplasm suggested the possibility of an endocrine neoplasm. IHC showed strong positive staining for synaptophysin, focal staining for chromogranin, and CD56. The negative TTF-1 and strong staining for PAX8 and RCC supported a metastatic tumour from the kidney, rather than a primary neuroendocrine carcinoma of the lung or metastasis from other sites. The strong staining of the lung tumour for CD10 suggested a metastasis from the primary PRCC component that had undergone neuroendocrine differentiation. Despite careful retrospective examination of the Hematoxylin and Eosin-stained sections of all blocks, the primary tumor in our case did not show morphological evidence of neuroendocrine differentiation. However, retrospective IHC detected focal positivity for synaptophysin in the PRCC component and these foci are presumed to be the source for the pulmonary metastases. Neuroendocrine differentiation of RCC (not primary neuroendocrine carcinoma of the kidney) is rare. Peckova et al. presented 18 ChRCCs with morphologic features suggestive of neuroendocrine differentiation. Four were confirmed by IHC [5]. Jung et al. reported 2 cases of MTSCC positive for chromogranin and synaptophysin [13]. Ronkainen et al. showed 1% of 152 primary RCC was positive for synaptophysin (including 1/131 CCRCC, 1/10 PRCC, and 0/4 ChRCC), 18% was positive for CD56 (including 23/128 CCRCC, 2/9 PRCC, and 1/4 ChRCC), and no case was positive for chromogranin [6].

Since the literature on RCC with neuroendocrine differentiation is based on case reports and small series, their prognosis and management are unclear [14]. However, knowledge of the possibility of neuroendocrine differentiation of renal carcinomas is helpful when diagnosing the nature and origin of metastatic carcinoma. Bressenot et al. reported a composite clear cell carcinoma and carcinoid tumor that metastasized to liver and bone [14]. Mokhtar et al. reported a ChRCC with neuroendocrine differentiation metastatic to a peri-hilar lymph node, the metastasis composed exclusively of the neuroendocrine component [3]. Two of four cases from Peckova's series had distant metastases [5]. Our patient developed lung and mediastinal nodal metastases 3 years after radical nephrectomy, which suggests that RCC with neuroendocrine differentiation is an aggressive tumor.
